# Sarcomatoid Intrahepatic Cholangiocarcinoma: A Rare and Aggressive Primary Liver Cancer

**DOI:** 10.7759/cureus.39520

**Published:** 2023-05-26

**Authors:** Eli A Zaher, Parth Patel, Ashwini Gotimukul, Hasan Sqour

**Affiliations:** 1 Department of Internal Medicine, Ascension Saint Joseph Hospital, Chicago, USA

**Keywords:** spindle cell lesion, cholangiocarcinoma, immunohistochemical stain, hepatocellular carcinoma (hcc), sarcomatoid intrahepatic cholangiocarcinoma

## Abstract

Sarcomatoid intrahepatic cholangiocarcinoma (S-iCCA) is a rare variant of primary liver cancer with a poor prognosis due to local aggressive expansion and frequent metastases. The pathogenesis remains unclear, but theories suggest epithelial-mesenchymal transition, biphasic differentiation of pluripotent stem cells, or sarcomatoid re-differentiation of immature multipotent carcinoma cells. Chronic hepatitis B and C, cirrhosis, and age above 40 are plausible contributors. Diagnosis of S-iCCA requires immunohistochemical evidence of both mesenchymal and epithelial molecular expression. Early detection and total resection are the current mainstay approach. We report a case of metastatic S-iCCA in a 53-year-old male with alcohol use disorder who underwent en bloc right hepatic lobectomy, right adrenalectomy, and cholecystectomy.

## Introduction

Sarcomatoid intrahepatic cholangiocarcinoma (S-iCCA) is one of the rarest variants of all primary liver cancers, accounting for less than 1% of cases [1]. The cells of the sarcomatoid component grow rapidly and frequently metastasize, contributing to the characteristic poor prognosis of the disease. While the pathogenesis remains unclear, theories have linked its origins with epithelial-mesenchymal transition, biphasic differentiation of pluripotent stem cells, or sarcomatoid re-differentiation of immature multipotent carcinoma cells derived from carcinoma cells [2-3]. The sarcomatoid component is occasionally seen in various types of epithelial tumors and is defined by immunohistochemical evidence of both mesenchymal and epithelial molecular expression. This characteristic sets it apart from intrahepatic cholangiocarcinoma with sarcomatoid transformation and carcinosarcoma, in which the sarcomatoid element expresses only epithelial or mesenchymal features, respectively [2]. Given the unclear utility of chemotherapy, surgical resection is the most common approach in management [4].

## Case presentation

**Figure 1:**
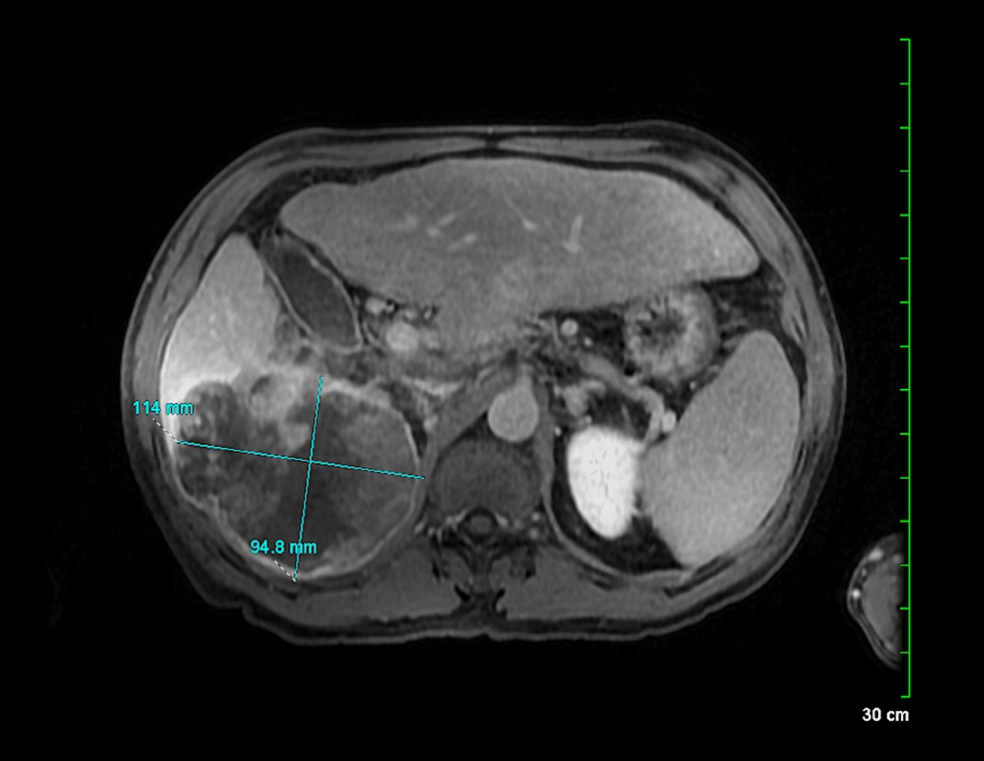
T1 MRI Liver Mass Protocol Magnetic resonance imaging shows a lobulated heterogeneously enhancing mass within the posterior and inferior right hepatic lobe abutting the right kidney.

Our patient is a 53-year-old Hispanic male with a history of alcoholic steatohepatitis and newly diagnosed diabetes mellitus who presented with three weeks of progressively worsening right upper quadrant (RUQ) abdominal pain and unintentional 15 lbs weight loss. Clinical examination demonstrated tenderness in the RUQ. CT abdomen showed a large solid mass in segment 8 of the liver with central necrosis and extracapsular expansion involving the upper pole of the right kidney. The liver was also enlarged but non-cirrhotic. Apart from paracaval and celiac lymphadenopathy, the other surrounding structures and organs were unremarkable. MRI abdomen with the liver protocol was performed for better visualization and revealed a lobulated, heterogeneously enhanced, infiltrating mass within the posterior and inferior aspects of the right hepatic lobe without evidence of metastasis (Figure 1).

**Figure 2:**
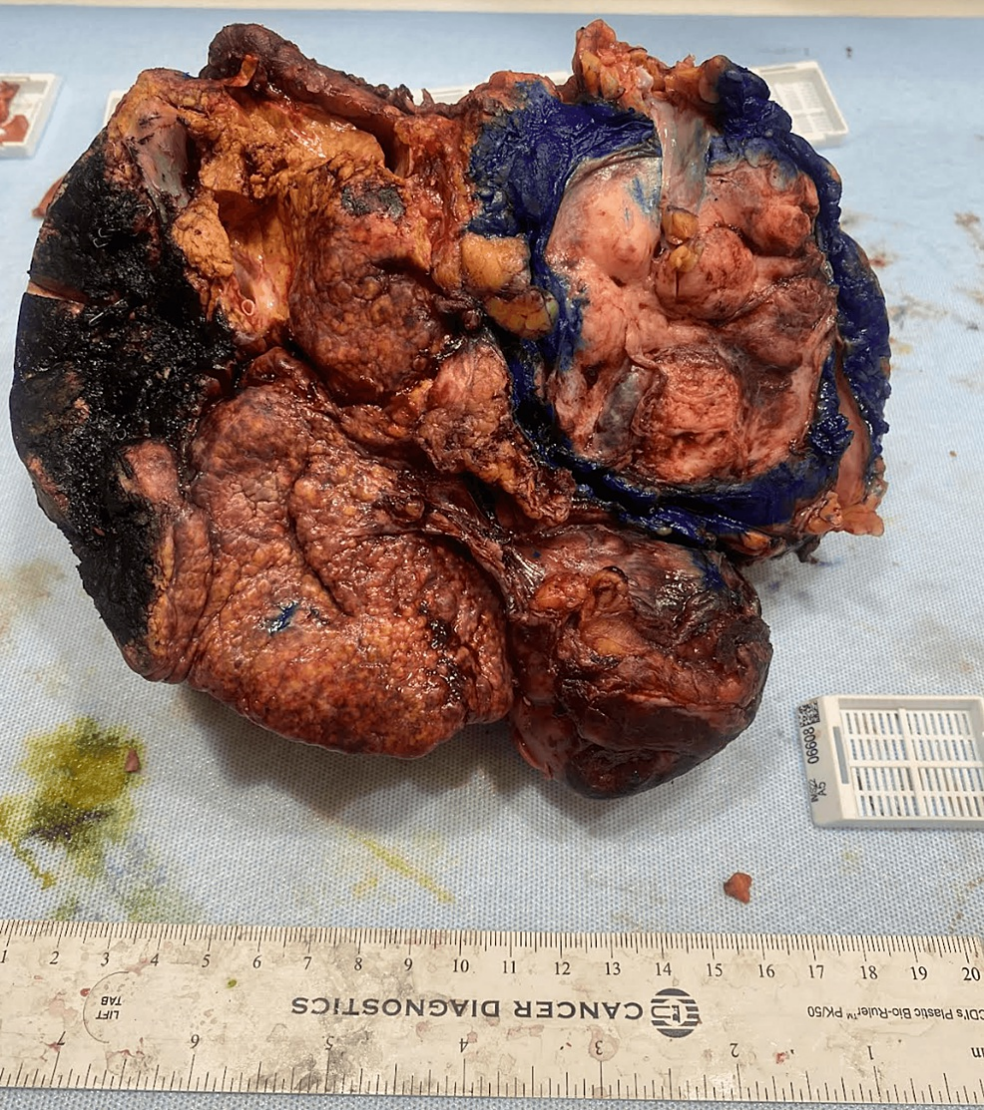
Gross Specimen Solid 17.8 x 13.5 x 10.2 cm mass retrieved from the right hepatic lobe.

Labs included mild transaminitis and a normal alpha-fetoprotein (AFP), cancer antigen 19-9 (CA19-9), and carcinoembryonic antigen (CEA). CT-guided core-needle liver biopsy was consistent with a malignant spindle cell neoplasm. Further staging with contrast-enhanced CT chest, upper endoscopy, and colonoscopy showed no evidence of malignant growth, placing the liver as the likely primary source of the tumor. Taken together, surgical resection was favored over chemotherapy. The patient underwent exploratory laparotomy with en bloc resection of the right hepatic lobe, right adrenalectomy, cholecystectomy, and resection of a diaphragmatic and peritoneal nodule. The confirmed tumor size after surgery was 17.8 x 13.5 x 10.2 cm (Figure 2).

**Figure 3:**
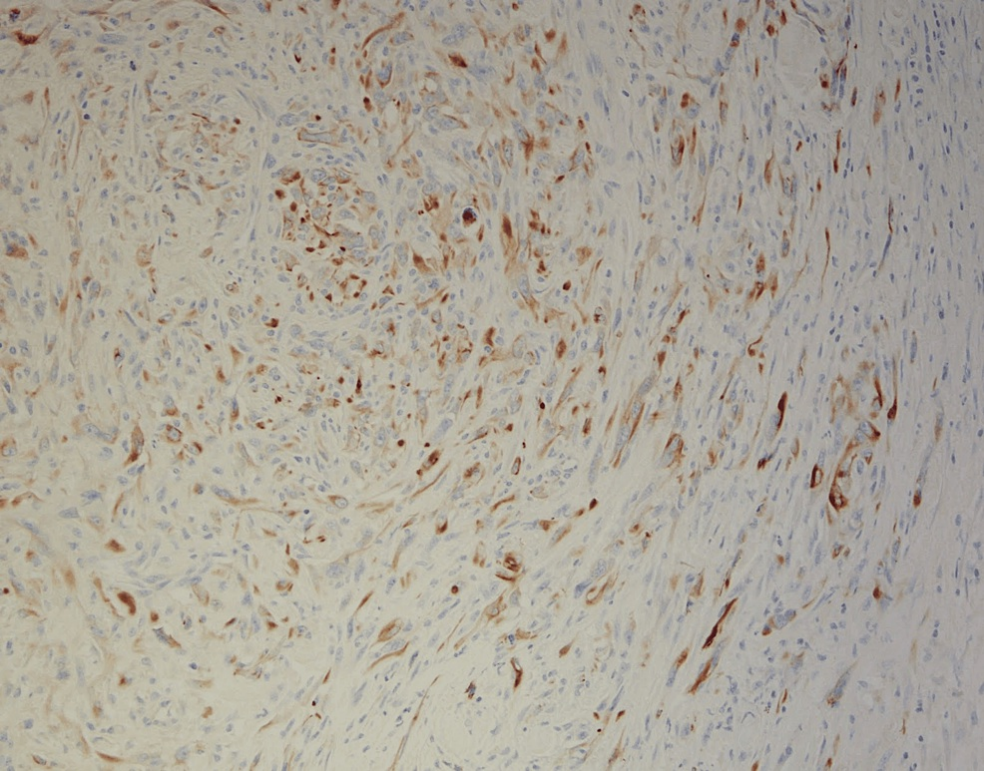
Immunohistochemical Stain for Vimentin and Caldesmon Highlighting sarcomatoid components.

**Figure 4:**
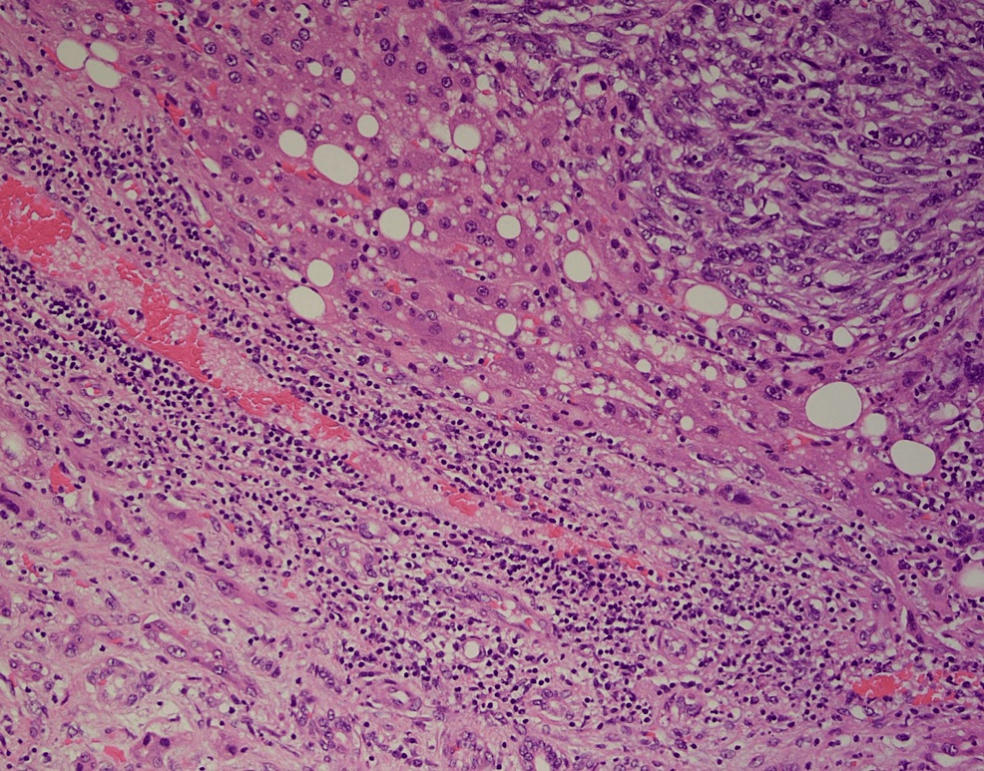
Staining for WSK Pankeratin and CK19 Wide-spectrum screening Pankeratin highlights carcinomatous components, while cytokeratin 19 highlights cholangiocarcinoma. Reticulin stain and hepatocyte paraffin 1 (HepPar1) show intact hepatic plates. WSK: wide-spectrum keratin, CK19: cytokeratin 19.

**Figure 5:**
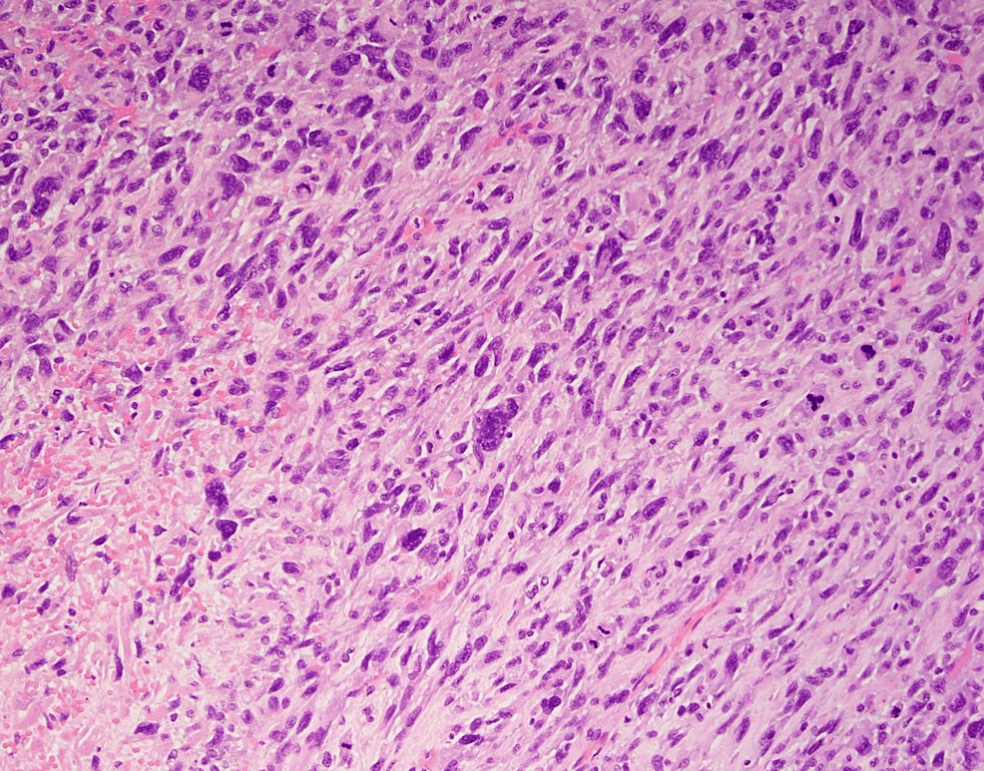
Extensive Necrosis

Following surgery, he was transferred for observation to the ICU due to intraoperative 1-liter blood loss with associated hypotension. Apart from referred right shoulder pain, his postoperative course was unremarkable. Surgical pathology was consistent with a grade 3 poorly differentiated sarcomatoid cholangiocarcinoma. The resected nodules from the diaphragm and right adrenal gland were also positive for sarcomatoid malignancy. The reported staging was pT4 N0. The sarcomatoid component was present on > 70% of the examined tissue with extensive necrosis. The tumor cells reacted positively with vimentin, caldesmon, and hepatocyte paraffin 1 (HepPar1) and negatively with wide-spectrum keratin (WSK) Pankeratin and smooth muscle actin (SMA) immunostains (Figures 3-5).

Considering the advanced stage and poor prognosis, the patient and his family opted for comfort care with hospice without a trial of radiotherapy or chemotherapy.

## Discussion

Sarcomatous change of primary liver tumors is mostly found in hepatocellular carcinoma, particularly following chemotherapy, radiofrequency ablation, transarterial chemoembolization, and percutaneous ethanol injection. However, sarcomatoid changes in cholangiocarcinoma, referred to as S-iCCA, are an extremely rare variant with unclear pathophysiology [1]. Per the World Health Organization (WHO) classification, S-iCCA is a cholangiocarcinoma with spindle cells that either resembles spindle cell sarcomas/fibrosarcomas or has features of a malignant fibrous histiocytoma [5]. Extensive intrahepatic expansion and frequent vascular metastases of the sarcomatous component contribute to a poor prognosis [6].

Risk factors are not well-established due to the rarity of the condition and limited research but are potentially age above 40 years, chronic hepatitis B, chronic hepatitis C, and liver cirrhosis [1,3]. While our patient was older than 40 years, he did not exhibit the latter risk factors. Data on alcohol abuse, which was present in our patient, as a risk factor for intrahepatic cholangiocarcinoma is conflicting [7-8]; however, no study linking it to S-iCCA was found in our literature search.

The current hypothesis regarding its pathogenesis includes (1) transformation or induced proliferation of epithelial carcinoma cells, also described as epithelial-mesenchymal transition, which plays a key role in sarcomatoid changes; (2) biphasic transformation of pluripotent stem cells to carcinoma or sarcoma; and (3) sarcomatoid re-differentiation of immature cancer cells with multipotent characteristics. The chronic inflammation associated with cirrhosis and viral hepatitis could explain their role in de-differentiation or biphasic differentiation and also the poor prognosis of S-iCCA [2-3].

The presence of a sarcomatoid component is an essential characteristic of S-iCCA, which is defined by immunohistochemical evidence of both mesenchymal and epithelial molecular expression. That is why, diagnosis of S-iCCA requires immunohistochemical features characterized by positivity in both epithelial (CK7, CK8) and mesenchymal markers (Vimentin) with histopathological features characterized by the concurrence of adenocarcinoma and sarcomatoid cells [2,9]. These histopathological characteristics of S-iCCA set it apart from intrahepatic cholangiocarcinoma with sarcomatoid transformation and carcinosarcoma, in which the sarcomatoid element expresses either only epithelial or only mesenchymal features, respectively [2]. Regarding our case, the sarcomatoid component was present in >70% of the examined tissue. Our case was also WNK Pankeratin and CK19 negative, which may be related to the de-differentiation of tumor cells during sarcomatous transformation.

Early detection and total resection is the current mainstay approach. The median survival time of patients with S-iCCA with and without surgery is 11 months and three months, respectively [10]. While adjuvant chemotherapy, especially gemcitabine, and cisplatin, may prolong survival, its effectiveness remains unclear [1,2,4-6]. Due to the rarity of the condition, there is not enough literature on immunohistochemical or histopathological factors affecting survival time.

## Conclusions

Sarcomatoid intrahepatic cholangiocarcinoma (S-iCCA) is a rare variant of primary liver cancer with a high metastatic potential and poor prognosis. The pathogenesis remains unclear, but it possibly arises from epithelial-mesenchymal transition, biphasic differentiation of pluripotent stem cells, or sarcomatoid re-differentiation of immature multipotent carcinoma cells. Diagnosis requires immunohistochemical evidence of both mesenchymal and epithelial molecular expression. Risk factors remain in debate, and our case puts into question the role of excessive alcohol use as a potential contributor to the pathogenesis of S-iCCA. It is most commonly managed by surgical resection, as chemotherapy remains unproven. Further research is required to develop effective treatment options for this aggressive disease.
